# Implementing in-vitro diagnostic point-of-care tests in community health care: how can we make this work?

**DOI:** 10.3399/bjgp25X741153

**Published:** 2025-03-28

**Authors:** Philip J Turner, Umasha Ukwatte, Ian Smith, Tim James, Paul Stephenson, Sarah Tonkin-Crine, Margaret Głogowska, Gail N Hayward

**Affiliations:** Nuffield Department of Primary Care Health Sciences, University of Oxford, Oxford; NIHR HealthTech Research Centre in Community Healthcare, Oxford Health NHS Foundation Trust, Oxford.; Nuffield Department of Primary Care Health Sciences, University of Oxford, Oxford; NIHR HealthTech Research Centre in Community Healthcare, Oxford Health NHS Foundation Trust, Oxford.; Department of Clinical Biochemistry, Oxford University Hospitals NHS Foundation Trust, Oxford.; Department of Clinical Biochemistry, Oxford University Hospitals NHS Foundation Trust, Oxford.; Nuffield Department of Primary Care Health Sciences, University of Oxford; Primary Care Respiratory Society, London.; Nuffield Department of Primary Care Health Sciences, University of Oxford, Oxford; NIHR HealthTech Research Centre in Community Healthcare, Oxford Health NHS Foundation Trust, Oxford.; Nuffield Department of Primary Care Health Sciences, University of Oxford, Oxford; NIHR HealthTech Research Centre in Community Healthcare, Oxford Health NHS Foundation Trust, Oxford.; Nuffield Department of Primary Care Health Sciences, University of Oxford, Oxford; NIHR HealthTech Research Centre in Community Healthcare, Oxford Health NHS Foundation Trust, Oxford.

## Introduction

In-vitro diagnostic point-of-care tests (POCTs) are performed across a broad range of sample types, with professional and self-administered POCTs gaining widespread familiarity during the SARS-CoV-2 pandemic. POCTs can rapidly inform clinical decisions, with potential benefits including the optimisation of disease management and referrals, reduction in healthcare costs, increased patient satisfaction, and better treatment adherence. Surveys have identified POCTs that GPs believed could be helpful and conditions where they considered that POCTs, if available, could support clinical decision making.^1^^,^^2^

Community-based health care is responsible for high volumes of activity (the majority of NHS contacts),^3^ dealing with largely undifferentiated patient populations, incorporating a high proportion of conditions that need minimal intervention or are self-limiting, but also low-prevalence, potentially serious conditions that require prompt, appropriate management. Community health care plays a fundamental role in disease prevention and the management of long-term conditions.

Implementation of POCTs in UK community health care does not currently align with the desire for POCTs reported previously.^1^^,^^2^ Evaluating evidence quality for the use of POCTs is crucial to support implementation decision making. While clinical performance is commonly studied, areas such as clinical effectiveness, cost-effectiveness, and broader impact are less frequently evaluated.^4^ A systematic review of implementation of 83 POCTs showed that clinical utility was of paramount importance for GPs, yet was rarely assessed.^5^ Limited community implementation of POCTs may be partly attributable to a lack of understanding of the elements required to achieve success. These include: GPs’ concerns about clinical utility; access to laboratory and diagnostic technology expertise; provision of funding, training, and support models; sound implementation strategies; and evidence-based monitoring.^6^^,^^7^

## The need for POCTs

Unmet need for POCTs can be identified through interviews and surveys,^1^^,^^2^^,^^8^^–^^10^ priority-setting partnership approaches,^11^ demand for laboratory-based diagnostics,^12^ or patterns of suboptimal prescribing.^13^ Sometimes these approaches highlight needs that cannot be met by available technology.^14^^,^^15^ Alternatively, value propositions for POCTs can be outlined by exploring the costs and benefits for key stakeholders. Value proposition frameworks for economic modelling can support this,^16^ in turn informing reimbursement decisions.^13^

## Clinical performance: addressing the concerns of community teams

### Are POCTs accurate?

To assure users of the accuracy of POCTs, evidence should be generated in the laboratory to ensure the test meets technical requirements; and by intended users in relevant clinical settings to assess robustness.^17^^,^^18^

Laboratory-based verification should include comparison between the POCT and clinical laboratory or reference methods on common or concurrent samples from the same patient, providing an indication of relative bias/inaccuracy. There is also a need to assess reproducibility (precision) through replicate analysis. This work should be laboratory led, and could be done once for a range of providers.

‘Real-world’ performance of POCTs in the intended clinical setting can deviate from manufacturers’ claims,^19^^,^^20^ hence the importance of establishing this before implementation. It can be influenced by factors related to sample collection and preparation, environmental conditions, and interpretation of results. Evaluations should take place in intended clinical settings, with target user groups, and on relevant patient populations.

Performance of POCTs is most important in the clinically relevant range for target use cases. For example, C-reactive protein above 20 mg/L can indicate the need for antibiotics in exacerbations of COPD;^21^ in this case, poor accuracy at levels greater than 200 mg/L is of little clinical consequence, but inaccuracy between 0 and 30 mg/L could affect patient outcomes.

## How can POCTs’ performance be maintained?

### Operator competence and training

Adequate training and continuous evaluation are required to prevent suboptimal operation of POCTs.^17^^,^^22^ Specific errors might include poor sample collection and processing, and delayed sample analysis. Initial training should be offered by manufacturers, with all training documented and traceable. Regular use of POCTs and continuous monitoring may improve operator skill retention and optimisation.^23^ Different training models are developed by organisations; in Oxford, a ‘hub and spoke’ structure was adopted by pathology services, with a team of ‘super-users’ training clinical operators.

### Quality control

Quality control (QC) processes provide users with confidence that a test is working as expected at a discrete timepoint. Internal controls are often integrated into POCT – for example, control lines on lateral flow tests. QC materials can be obtained from manufacturers or from independent suppliers. In larger organisations, QC materials can be used for batch acceptance testing of new consumables or stand-alone tests.

### External quality assurance scheme enrolment

External quality assurance (EQA) schemes (such as UK NEQAS and WEQAS) provide operators with independent information on the accuracy of diagnostic instruments by comparison with similar machines. These schemes offer broad but not comprehensive coverage of POCT-relevant biomarkers.

## How can results of POCTs be captured?

The accurate recording of test results in the electronic patient record (EPR) is vital to support immediate and subsequent clinical decisions and governance.

Modern instrument-based POCTs commonly incorporate data connectivity, facilitating transfer of results to the EPR. Middleware software provides a link between many POCTs and EPRs, and allows central management and reporting. Deployment of middleware is unlikely to be practical for individual community sites but could be used within networks or where a hospital laboratory acts as the community provider of testing services. Middleware solutions can be used to support good governance and EQA.

Disposable POCTs achieved global use in the SARS-CoV-2 pandemic, but accurate reading, interpretation, and entry of results into the EPR can be challenging. Hardware and software solutions that interpret and securely transmit results to patient records are available or are in development.

## Practical considerations when choosing POCTs

### Management of consumables

Storage and management requirements for POCTs consumables can vary. Shelf-life is important, particularly where assays are used infrequently. It may be possible to split batches of consumables across sites within an organisation, but this is not always possible, as cartons from some manufacturers carry machine-readable information, essential for the functioning of analysers. Refrigeration requirements add complexity.

### Operating environment

POCTs should only be operated within the settings designated by the manufacturer. Upper and lower limits of operating temperature and humidity could restrict the settings in which devices can be operated – for example, in homes or ambulances.

### Device footprint, weight, and capability

Space availability for POCTs may be a limiting factor, which could bias choice towards those which are disposable or have a small footprint. Clinicians should have easy access to POCTs, otherwise they are likely to be overlooked or avoided during consultations.^24^ Weight must be considered if a device will be transported. Multi-test platforms offer benefits in this regard, by offering a selection of different assays or panels on a single device, and advantages in terms of stock control and training.

### Biosafety

Operation of POCTs could constitute an additive biosafety risk above that posed by the unwell patient. The emergence of SARS-CoV-2 heightened awareness of these risks, and initiated work by the UK Health Security Agency (UKHSA) on the capacity of diagnostic test buffers and media to inactivate the virus.^25^

## Implementation strategy

### Behavioural change

Organisation-wide adoption of POCTs requires a change in behaviour at individual and system levels.^26^^,^^27^ POCTs are designed to inform clinicians’ decision making, leading them to act differently (where appropriate) compared with how they would without POCTs. For POCTs to have an impact on practice, clinicians must use the test, understand it, and act on the result. This requires motivation, capability, and opportunity.^28^ Motivation requires that clinicians’ concerns about clinical utility and POCT accuracy have been addressed,^6^^,^^7^^,^^9^ and that clinicians believe POCTs will positively influence practice, in terms of helping to manage patients without increasing workload, and lead to positive patient outcomes, for example, patients being happy and satisfied with their care.^29^ Capability to use and interpret tests comes from training. Clinicians can often identify opportunities to use POCTs if they hear that peers, working in similar environments, have had a positive experience with them.^30^

### Clinical training

Clinicians will need training on test interpretation in the context of the patient’s presentation. POCTs are particularly beneficial when there is clinical uncertainty; if the clinician is already confident in their treatment decision, they will be unlikely to use POCTs. This can be a problem for promoting antibiotic stewardship in common conditions where clinicians experience uncertainty infrequently despite overprescribing antibiotics.^31^ Here there is an argument for use of POCTs to become compulsory for all presentations so that clinicians become aware of the discrepancy between their prescribing and the illness severity of their patients. However, if a result differs from what is expected from the clinical presentation, the clinician may override the result and treat the patient differently. Management decisions are not solely influenced by clinical factors, as social and other factors (such as patient preference and the length of consultation) can determine how a clinician chooses to treat a patient.^30^ Patient influence on decisions is also important; a clinician might use POCTs to convince a patient of a treatment plan because they believe the patient will only trust the technology.^10^ While this approach may be useful, it can increase expense, risks over-reliance on POCTs at the cost of clinicians’ communication skills, and may encourage future consultations for self-limiting illness.^10^ Providing broader training on incorporation of POCTs into workflows and how to discuss results with patients can help to address these factors, thereby optimising patient management and increasing patient satisfaction.^24^^,^^32^

### Organisational change

Clinicians should have clear local guidance on how POCTs will be incorporated into workflows and should be confident which patients they should be testing to avoid misuse.^26^ Sites may choose to triage patients to specific staff, trained to use a POCT (meaning that clinicians get faster and more skilled at using the test because of frequent use); alternatively, they may have a POCT available for any clinician to use.^24^ Site champions could support implementation by sharing evidence on benefits, example case studies, and addressing concerns about use.^24^^,^^32^

Nationally, pathology and clinical engineering technology expertise will be required,^33^ as well as input from community care clinicians who are already using POCTs. This is likely to be delivered most effectively at integrated care board (ICB) level.

### Funding

International evidence for the cost-effectiveness of POCTs in community care can be convincing.^34^^–^^38^ However, decision makers may still require evidence applicable to their own settings.^26^ Start-up funding will be required for widespread implementation in the UK. It will be essential to fund pathology and clinical engineering technology expertise appropriately, and funding incentives will be needed for ICBs, primary care networks, and/or GP practices.^7^^,^^13^^,^^39^ It is important to recognise that cost savings may not be realised by those carrying out testing, but may be experienced elsewhere in the system.

### Environmental impact

The global environmental impact of healthcare provision is recognised^40^ and the UK has taken legislative steps to address this within the NHS. Plastics produced from non-renewables are commonly used in the manufacture of POCTs, and their disposal contributes to greenhouse gas emissions.^41^ It is important that healthcare providers take an environmental cost–benefit approach to the implementation of new tests.

### Practical consideration points for implementation

- POCT introduction and ongoing management can be complex, so partnering with a pathology service where possible can alleviate the burden of logistics, maintenance, and governance;
- All the potential POCTs available should be reviewed to identify the options that will fit best with the way the clinician delivers care, as there can be variability in portability, maintenance requirements, and performance.
- Experience indicates that clinics adopting new tests do best where an enthusiastic team member takes ownership of POCTs and provides ongoing support to other team members.
- It should be ensured that initial POCT training is in person rather than online; qualitative work suggests that this is far preferable to end-users and is more likely to inspire confidence.
- It should be considered whether a new test will really add value over current practice, and how the clinician might evaluate this once it is in place.
- An ongoing relationship with the test manufacturer or laboratory for uninterrupted supply of consumables and timely intervention should be ensured when things go wrong.

## Conclusion

Patients and healthcare systems can benefit from treatment decisions informed by prompt results from appropriate POCTs. Currently, the UK lacks widespread implementation of potentially transformative POCTs in community settings. By applying the considerations outlined above to new implementation initiatives, we are likely to improve the likelihood of successful adoption and sustained use.
